# Traditional Chinese medicine constitution and sarcopenia: a cross-sectional study

**DOI:** 10.3389/fpubh.2024.1368933

**Published:** 2024-07-24

**Authors:** Chi Wang, He Zhang, Xin Nie, Fei Ding, Qianhui Liu, Lisha Hou, Yiping Deng, Wenbin Ye, Jirong Yue, Yong He

**Affiliations:** ^1^Department of Laboratory Medicine, West China Hospital, Sichuan University, Chengdu, Sichuan, China; ^2^Sichuan Clinical Research Center for Laboratory Medicine, Chengdu, Sichuan, China; ^3^Clinical Laboratory Medicine Research Center of West China Hospital, Chengdu, Sichuan, China; ^4^Department of Geriatrics and National Clinical Research Center for Geriatrics, West China Hospital, Sichuan University, Chengdu, Sichuan Province, China; ^5^Xiamen Hospital of Traditional Chinese Medicine, Beijing University of Chinese Medicine, Xiamen, Fujian, China

**Keywords:** sarcopenia, middle-aged and older, traditional Chinese medicine, Yin-deficiency constitution, propensity score matching

## Abstract

**Objective:**

Sarcopenia is a gradually advancing systemic disorder affecting skeletal muscles, primarily distinguished by diminished muscle mass and functional decline. As of present, a universally accepted diagnostic criterion for sarcopenia has yet to be established. From the perspective of the constitution theory in traditional Chinese medicine (TCM), the Yin-deficiency constitution is believed to have a significant correlation with the development of sarcopenia. The primary objective of this study was to examine the potential association between sarcopenia and Yin-deficiency constitution.

**Methods:**

The present study is a cross-sectional analysis. The Asian Working Group for Sarcopenia (AWGS) recommended a diagnostic criterion for sarcopenia. A total of 141 participants over 50 years of age were diagnosed with sarcopenia. To determine the constitution of each patient, classification and determination standards were used in traditional Chinese medicine. In this study, a combination of logistic regression and propensity score matching (PSM) was employed to analyze a dataset comprising 1,372 eligible observations. The diagnostic efficacy of the test in distinguishing sarcopenia was assessed through receiver operating characteristic (ROC) curve analysis.

**Results:**

The relationship between Yin-deficiency constitution and sarcopenia was examined using logistic regression analysis. In the crude model, the odds ratio (OR) was found to be 3.20 (95% confidence interval [CI]: 1.70–6.03). After adjusting for various confounding factors, including gender, sex, 6 m walking test/(m/s), SMI, and maximum grip strength/kg, the OR increased to 9.70 (95% CI: 3.20–69.38). The associations between seven other biased traditional Chinese medicine (TCM) constitutions and sarcopenia were not found to be statistically significant in the fully adjusted model. The propensity score matching (PSM) analysis yielded consistent results with the logistic regression analysis. Receiver operating characteristic (ROC) curve analysis showed that the AUC of the Yin-deficiency constitution combined with age and gender reached 0.707.

**Conclusion:**

Among the nine TCM constitutions examined, the Yin-deficiency constitution demonstrates an independent association with sarcopenia. Yin-deficiency constitution may serve as a potential risk factor for the development of sarcopenia. To establish a causal relationship, further experimental investigations are warranted. The diagnostic performance of sarcopenia is effectively demonstrated by the Yin-deficiency constitution combined with age and gender.

## Introduction

Sarcopenia is mainly due to continuous skeletal muscle loss and decreased strength and function, which is manifested by reduced muscle strength and decreased muscle mass (1, 2). As a result of aging or chronic disease, sarcopenia is divided into primary sarcopenia and secondary sarcopenia. This study focuses on primary sarcopenia, which refers to the senile syndrome where various mechanism changes caused by body aging further lead to the loss of skeletal muscle mass and the decline of physical strength (3, 4). The concept of sarcopenia was initially introduced by Rosenberg in 1989 (5). In 2010, the European Working Group on Sarcopenia (EWGSOP) published the first consensus on sarcopenia, which characterizes it as a syndrome prevalent in old age, marked by a decline in muscle mass, muscle strength, and/or physical performance (6). Building upon this definition, the Asian Sarcopenia Working Group (AWGS) adopted the same conceptualization in 2014 while also delineating the specific boundaries within Asia (7, 8). As of 2019, the Asian Working Group for Sarcopenia (AWGS) has redefined sarcopenia to include possible sarcopenia, sarcopenia, and severe sarcopenia (9). In addition to genetic, age, and other factors, sarcopenia is associated with a multitude of chronic diseases, resulting in a complex etiology with numerous influencing factors (3). The clinical manifestations of muscle atrophy and strength decline in sarcopenia align with the concept of Wei syndrome in traditional Chinese medicine (10). This study attempts to analyze the correlation between the two from the perspective of traditional Chinese medicine constitution related to body function.

The concept of body constitution, which is a fundamental aspect of traditional Chinese medicine (TCM), is intricately linked to human health and the development of diseases (11, 12). Various constitutions exhibit distinct susceptibilities and inclinations toward different types of tumors. Consequently, the identification and modification of one’s constitution can significantly impact the prognosis of disease regression (13, 14). Traditional Chinese medicine constitution is derived from the Huangdi Neijing and has been developed by doctors of various generations of medicine. Professor Wang Qi divided it into nine types, comprising one constitution type characterized by balance (Neutral) and eight constitution types exhibiting bias (Qi-deficiency, Yang-deficiency, Yin-deficiency, Blood-stagnation, Qi-stagnation, Damp-heat, Phlegm-damp, and Special diathesis constitutions) (15, 16).

Our results showed that Yin deficiency constitution, one of the biased constitutions, was directly related to the diagnosis of sarcopenia by Pearson’s correlation (Supplementary Table S1). Yin-deficiency constitution refers to a physiological condition characterized by a depletion of bodily fluids, essence, and blood, as well as a lack of moisture and nourishment resulting from the persistent impact of both innate predisposition and external factors (17). The Yin-deficiency constitution primarily exhibits the following features: (1) physical attributes encompassing a slender and lean physique; (2) psychological traits characterized by liveliness, extroversion, irritability, and high activity levels; and (3) prevalent clinical manifestations include warm extremities, palpitations, parched mouth and throat, a red tongue with minimal coating, dry eyes, impaired vision, and parched skin (18, 19). Yin-deficiency constitution is intricately linked to the aging process and serves as a predisposing factor for various diseases, notably osteoporosis, which exhibits a high prevalence among individuals with this constitution (17, 20). This parallels the age-related manifestations of sarcopenia. Furthermore, individuals with Yin deficiency constitution display varying degrees of endocrine and immune dysfunction, while existing literature has established a correlation between abnormal metabolic function and sarcopenia in patients (19, 21). The clinical manifestations of sarcopenia bear striking resemblance to those of Yin-deficiency constitution, leading us to hypothesize that the latter serves as the underlying pathology for sarcopenia. However, no empirical evidence from relevant studies exists to support this claim. To establish a correlation between the two, we have undertaken a cross-sectional study.

## Methods

### Data collection

The cross-sectional analysis of the current research incorporates baseline data obtained from the WCHAT study, which was initiated from March to April 2022 and consists of 1,372 people aged 50 or older in the city of Xiamen. The study’s exclusion criteria comprised the following: (1) cognitive impairment; (2) recent malignancy history; (3) incomplete laboratory measurement data; and (4) medical history of plate, pacemaker, and cardiac stent implantation existed. Among the participants, 141 individuals were diagnosed with sarcopenia. This study was approved by the Ethics Committee of the West China Hospital of Sichuan University. All participants demonstrated a willingness to participate in the study and provided informed consent.

### Assessment of sarcopenia

The AWGS 2019 diagnostic criteria, which incorporate assessments of muscle mass loss, muscle strength loss, and physical performance loss, were employed to diagnose sarcopenia in this study, as they have been widely utilized in Asia. Muscle strength was measured twice using a dynamometer (EH101; Camry, Zhongshan, China) with the dominant hand, and the highest value was utilized for analysis. Low muscle mass was defined as an appendicular skeletal muscle mass index (ASMI) of less than 7.0 kg/m^2^ for males and less than 5.7 kg/m^2^ for females, as determined by bioimpedance analysis. Additionally, low muscle strength is also defined as handgrip strength values of less than 28 kg and less than 18 kg for men and women, respectively, according to the AWGS. The evaluation of physical ability is recommended through the use of the 5 m walking test, with a speed of less than 1.0 m/s and an SPPB score of ≤9, as suggested by the AWGS.

### Assessment of TCM constitution

The diagnostic criteria for TCM constitution, namely, the “TCM Constitution Classification Criteria” and the “TCM Constitution Classification Scale of 9 Basic Constitutions,” have been developed by Professor Wang and are presently acknowledged in the field. A total of 1,372 participants were recruited from various communities during the period of March to April 2022. The assessment of TCM constitution was conducted using a standardized questionnaire endorsed by the China Association for Traditional Chinese Medicine (Supplementary material). Questionnaire data are collected by professionals through personal, one-on-one interviews conducted in a face-to-face manner. The questionnaire consists of 60 items, with nine subscales containing 7–8 items each, interspersed irregularly. Each item is answered using a Likert scale, ranging from “no” to “always,” with scores ranging from 1 to 5. The original score for each subscale is calculated by summing the scores for each item and then converted into a conversion score using the formula [(original score – number of subscale items)/(number of subscale items × 4) × 100]. The conversion score for each subscale ranges from 0 to 100 points. The conversion score of the neutral constitution scale was ≥60 points, and the conversion score of the other 8 biased constitution scales was <30 points. A biased constitution was diagnosed if the conversion score for any constitution was equal to or greater than 40 points.

### Statistical analysis

Measurement data of continuous variables are presented as the mean ± SD (normally distributed) and the median (interquartile range) for non-normally distributed and categorical variables are expressed as frequencies with percentages. Differences between groups were tested by *t* test for normally distributed data, Mann Whitney *U* test for skewed continuous variables and the chi-square test was used for categorical variables. Binary regression was used to evaluate the association between the nine traditional constitutions and sarcopenia. We presented the findings of unadjusted and minimally adjusted analyses alongside fully adjusted analyses in a simultaneous manner. To ensure the robustness of the results, we categorized participants based on their Yin-deficiency constitution and employed the PSM (propensity score matching) technique for participant matching. The caliper was set at 0.01, and a 1:1 ratio was maintained while matching for sex, age, 6 m walking test/(m/s), SMI, and maximum grip strength/kg. Next, logistic regression was employed to compute the odds ratio and establish a 95% confidence interval. A *p* value <0.05 was considered to indicate statistical significance. The diagnostic efficacy of the test in distinguishing sarcopenia was assessed through receiver operating characteristic (ROC) curve analysis and ROC comparison analysis in MedCalc v19.0.7.

## Results

### Characteristics of the study cohort stratified by sarcopenia

Among 1,372 participants, 141 (10.3%) were diagnosed with sarcopenia. Table 1 presents the baseline characteristics of the older people categorized by sarcopenia. The average age of the participants was 64.77 ± 7.29 years old, and approximately 24.85% of them were male. Patients with sarcopenia had higher values in the sit-to-stand five times test but lower values in the 6 m walking test, SMI and Maximum grip strength than the healthy controls (*p* < 0.001). Regarding the distribution of TCM constitution, compared with the non-sarcopenia group, people with sarcopenia had a higher proportion of biased constitution and a lower proportion of balanced constitution (neutral). Meanwhile, there were no differences between the groups with and without sarcopenia regarding the Phlegm-damp, Damp-heat, and Special diathesis constitution.

**Table 1:** Characteristics of the study cohorts.

	Total (*n* = 1,372)	No sarcopenia (*n* = 1,231)	Sarcopenia (*n* = 141)	*p*
Gender, male (*n*, %)	341 (24.85)	302 (24.53)	39 (27.66)	0.416
sit-to-stand five times/s	9.52 ± 2.12	9.23 ± 1.68	12.10 ± 3.42	<0.001
6 m walking test/(m/s)	1.18 ± 0.22	1.20 ± 0.21	0.99 ± 0.20	<0.001
SMI	6.46 ± 0.84	6.56 ± 0.80	5.59 ± 0.57	<0.001
Maximum grip strength/kg	26.2 (23.13–30.6)	26.3 (23.6–31)	21.9 (19.1–25.15)	<0.001
Age (years, mean ± SD)	64.77 ± 7.29	64.24 ± 7.06	69.48 ± 7.59	<0.001
Neutral constitution	1,098 (80.03)	1,015 (82.45)	83 (58.87)	<0.001
Qi-deficiency constitution	65 (4.74)	55 (4.47)	10 (7.09)	0.165
Yang-deficiency constitution	164 (11.95)	121 (9.83)	43 (30.50)	<0.001
Yin-deficiency constitution	55 (4.01)	41 (3.33)	14 (9.93)	<0.001
Phlegm-damp constitution	28 (2.04)	24 (1.95)	4 (2.84)	0.480
Damp-heat constitution	12 (0.87)	9 (0.73)	3 (2.13)	0.092
Blood-stagnation constitution	65 (4.74)	56 (4.55)	9 (6.38)	0.332
Qi-stagnation constitution	31 (2.26)	26 (2.11)	5 (3.55)	0.278
Special diathesis constitution	9 (0.66)	9 (0.73)	0 (0.00)	0.308

Mean skeletal muscle mass index, SMI.

### Relationship between traditional Chinese medicine constitutions and sarcopenia

Multivariate logistic regression analysis was conducted to examine the relationship between TCM body constitutions and sarcopenia (Table 2). TCM constitutions were considered as the independent variables, while sarcopenia was regarded as the dependent variable. The crude model showed that without adjusting for any confounding variables, the Yin-deficiency constitution (OR = 2.67, [95% CI: 1.04–6.85]), the Neutral constitution (OR = 0.31, [95% CI: 1.04–6.85]) and the Yang-deficiency constitution (OR = 4.03, [95% CI: 2.69–6.03]) were identified as risk factors for sarcopenia. However, based on the findings of univariate analysis, adjusting for gender, age, 6 m walking test/(m/s), SMI, and maximum grip strength/kg by stepwise logistic regression, in the fully adjusted model, there was still statistical significance (*p* < 0.001) in Yin-deficiency (Table 3).

**Table 2:** Relationship between traditional Chinese medicine constitutions and sarcopenia in different models.

	Crude model		Minimally		Fully	
	OR (95%CI)	*p*	OR (95%CI)	*p*	OR (95%CI)	*p*
Neutral constitution	0.31 (0.21–0.44)	<0.001	0.47 (0.28–0.78)	0.004		
Qi-deficiency constitution	1.63 (0.81–3.33)	0.169				
Yang-deficiency constitution	4.03 (2.69–6.03)	<0.001				
Yin-deficiency constitution	3.20 (1.70–6.03)	<0.001	2.67 (1.04–6.85)	0.041	9.70 (3.20–69.38)	<0.001
Phlegm-damp constitution	1.47 (0.50–4.29)	0.483				
Damp-heat constitution	2.95 (0.79–11.03)	0.108				
Blood-stagnation constitution	1.43 (0.69–2.96)	0.334				
Qi-stagnation constitution	1.70 (0.64–4.51)	0.283				
Special diathesis constitution	-	-				

Crude model adjusted for none. Minimally adjusted model adjusted for gender, age, SMI. Fully adjusted model adjusted for gender, age, 6 m walking test/(m/s), SMI, and maximum grip strength/kg. OR, odds ratio; CI, confidence interval.

**Table 3:** Characteristics of the non-Yin-deficiency and Yin-deficiency constitution groups after matching.

	Non-Yin-deficiency constitution (*n* = 51)	Yin-deficiency constitution (*n* = 51)	*p*
Gender, male (*n*, %)	45 (88.24)	44 (86.27)	0.767
sit-to-stand five times/s	9.36 ± 1.62	10.01 ± 2.15	0.088
6 m walking test/(m/s)	1.29 ± 0.20	1.19 ± 0.20	0.015
SMI	6.37 ± 0.74	6.25 ± 0.69	0.392
Maximum grip strength/kg	26.92 ± 5.99	25.34 ± 6.14	0.191
Age (years, mean ± SD)	64.27 ± 8.20	65.20 ± 7.72	0.56

### Sensitivity analysis

Furthermore, the PSM analysis method was employed to successfully match 51 pairs of participants in both the Yin-deficiency constitution group and the non-Yin-deficiency constitution group. There was no statistically significant difference between the Yin-deficiency constitution group and the non-Yin-deficiency constitution group in relation to sex, age, SMI, or maximum grip strength/kg. Upon adjusting for confounding variables, the odds ratio (OR) of the Yin-deficiency constitution group was determined to be 15.39 (1.92–123.46) (Table 4) and exhibited a prevalence of sarcopenia at 23.53%, whereas the non-Yin-deficiency constitution group had a prevalence of 1.96%, aligning with the findings of the multivariate logistic analysis.

**Table 4:** Prevalence of sarcopenia in the non-Yin-deficiency and Yin-deficiency constitution groups after matching among 1,372 participants.

	Yin-deficiency constitution		OR	
	No	Yes	OR (95%CI)	*p*
Sarcopenia (*n*, %)	1 (1.96)	12 (23.53)	15.39 (1.92–123.46)	0.010

OR, odds ratio; CI, confidence interval.

### The predictive value of indicators for sarcopenia

**Figure 1:**
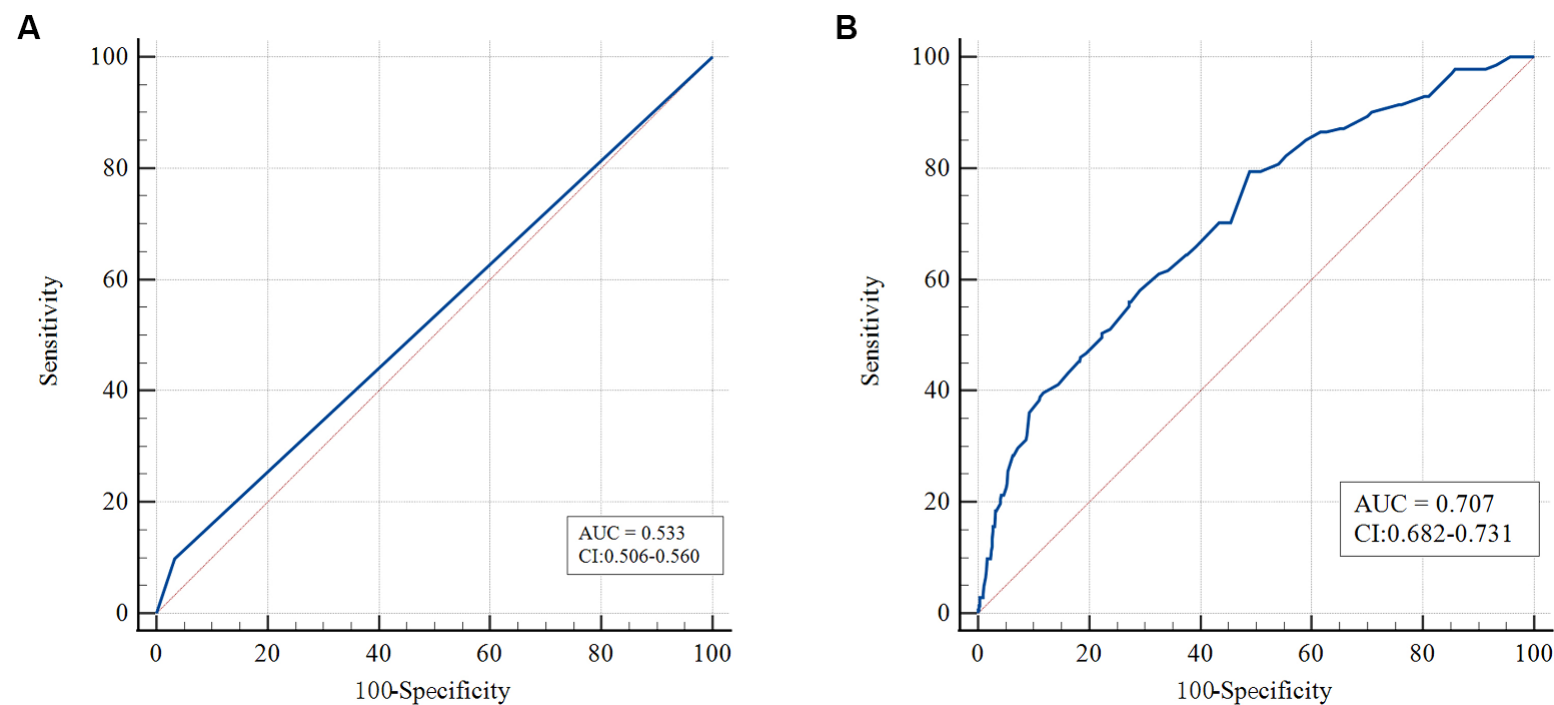
Receiver operating characteristic curves of TCM constitution indicators in sarcopenia. **(A)** ROC curve analysis of Yin-deficiency constitution with AUC. **(B)** ROC curve analysis of comprehensive diagnosis between Yin-deficiency constitution and gender and age.

Meanwhile, we assessed the diagnostic performance of the Yin-deficiency constitution group reflecting the low muscle mass in the sarcopenia cohort by the area under the curve (AUC) (Figure 1). We found that the AUC of the Yin-deficiency mixture reached 0.707, which exhibits good diagnostic performance for sarcopenia.

## Discussion

Sarcopenia in older adults is linked to a range of unfavorable outcomes, such as restricted physical functioning, falls, heightened risk of fractures, and elevated mortality rates (22, 23). Presently, the prevailing approach to managing sarcopenia in Western medicine involves exercise and nutritional supplementation (24). However, the efficacy of training interventions varies due to disparities in exercise modalities, duration and intensity, the absence of universally standardized protocols tailored to individuals, and contentious recommendations regarding nutrient intake (25). In the present study, we investigated the association of TCM constitution and sarcopenia. Our study found that people with sarcopenia have a higher proportion of biased constitution. Meanwhile, after adjusting for confounding variables, such as gender, age, 6 m walking test/(m/s), SMI, and maximum grip strength/kg, the logistic regression found that the Yin-deficiency constitution was independent of sarcopenia. In addition, the sensitivity analysis yielded consistent outcomes when employing the PSM method to match the participants and evaluated the predictive performance through the AUC, thereby providing further evidence for the stability of the relationship between the Yin-deficiency constitution and sarcopenia.

The development of sarcopenia is characterized by the degeneration of skeletal muscle throughout the body, a phenomenon that can be classified under the “Wei syndrome” in Chinese medicine (13, 26). This is defined as a clinical condition resulting from diminished blood supply due to external or internal factors, leading to a gradual deprivation of nourishment to the muscles and subsequent manifestation of limb weakness, muscle wasting, and potentially paralysis (27, 28). Sarcopenia mainly shows reduced skeletal muscle mass with muscle mass, decreased strength and activity impairment, which are very similar in terms of pathogenesis and clinical characteristics (29). As one of the important components of TCM, TCM constitution is closely related to human health and the occurrence of diseases (30). TCM acknowledges that TCM constitution plays a fundamental role in the initiation, advancement, and advancement of diseases (31). Thus, individual constitution affects the human body’s susceptibility to pathogenic factors and determines whether the individual is sick and the severity of the disease (32). Yin-deficiency constitution is one of the types of traditional Chinese medicine constitution. Innate inherited and acquired on the basis of sperm blood fluid in the body Yin fluid deficiency formed by Yin-deficiency internal heat as the main feature constitution condition (33). It is the constitution basis for forming many diseases (19, 34). Constitution not only determines whether the human body occurs after that, but also determines the tendency of the onset after the disease (35). That determines the susceptibility syndrome. Syndrome is a unique concept in traditional Chinese medicine. The syndrome combines the pathogenic factors and the body response. And the body reaction and the individual constitution are inseparable (36). Similarly, in the process of disease in different stages, which is also inseparable from physical factors (37). To Chinese medicine dialectical point of sarcopenia belongs to the category of Wei syndrome, and the nature of Wei syndrome is the essence of the excess in superficiality and deficiency in origin, and the deficiency includes the deficiency of Qi, Yin and Yang, which is inevitably linked to the Yin-deficiency constitution, because there is a state of deficiency of essence, blood and body fluid in the pathological manifestations (27, 38, 39). The correlation between Yin-deficiency and sarcopenia may be due to the common pathological basis of both. Clinical studies have shown that Yin-deficiency is associated with decreased neurocognitive function in older people, mainly affecting the visuospatial dimension (35). Yin-deficiency constitution is also a constitution type closely associated with aging (20). This molecular mechanism potentially stems from the involvement of five genes, namely, TAK1, NFKBIA, CCL4, BCL2A1, and IL-8, which are closely implicated in the NF-kappaB signaling pathway and the aging process (20). This may be due to congenital physical factors and acquired lifestyle; subsequently, the organism enters a Yin-deficiency constitution, leading to differences in the expression of these genes and eventually the development of sarcopenia.

At present, there are no clinical studies on TCM constitution and sarcopenia. To our knowledge, this is also the first study to predict the risk of sarcopenia using TCM constitution. The current clinical diagnosis of sarcopenia is inconsistent, and the treatment is complex. Currently, in terms of prevention, TCM has mature criteria for determining body constitution, which are inexpensive and easily accessed for sarcopenia in the clinical setting. Specifically, TCM interventions, including diet, exercise, medicine and acupuncture, may provide new clinical ideas. Certainly, there are obvious complexities and limitations inherent in this study. (1) The results of this paper can only demonstrate the correlation between Yin-deficiency constitution and sarcopenia but cannot draw causal conclusions. (2) The percentage of Yin-deficiency constitution is relatively low in the recruited people and the result may be biased. (3) This is a cross-sectional study with geographical restrictions. (4) Climate, diet, lifestyle habits, socioeconomic status are not taken into account. (5) Other unknown confounding variables still need to be adjusted. The sample of this study is mainly concentrated in Xiamen, and the inclusion indicators are not comprehensive, so it is necessary to conduct comprehensive and in-depth exploration by multi-center, larger sample, longer time span and more relevant indicators in the future.

## Conclusion

Among the nine TCM constitutions, the Yin-deficiency constitution is independently associated with sarcopenia. Therefore, we postulated that the Yin-deficiency constitution may serve as a potential risk factor for sarcopenia.

## Data availability statement

The raw data supporting the conclusions of this article will be made available by the authors, without undue reservation.

## Ethics statement

This study was approved by the Medical Ethics Committee of West China Hospital of Sichuan University (ethics approval No. 2017445), and the registration number is ChiCTR 1800018895. The studies were conducted in accordance with the local legislation and institutional requirements. The participants provided their written informed consent to participate in this study.

## Author contributions

CW: Data curation, Investigation, Writing – original draft, Writing – review & editing. HZ: Conceptualization, Formal analysis, Funding acquisition, Writing – review & editing. XN: Data curation, Supervision, Validation, Writing – review & editing. FD: Methodology, Resources, Validation, Writing – review & editing. QL: Formal analysis, Validation, Writing – review & editing. LH: Validation, Visualization, Writing – review & editing. YD: Methodology, Validation, Writing – review & editing. WY: Data curation, Supervision, Writing – review & editing. JY: Project administration, Supervision, Writing – review & editing. YH: Data curation, Funding acquisition, Project administration, Resources, Supervision, Writing – review & editing.

## References

[1] Damluji AA Alfaraidhy M AlHajri N Rohant NN Kumar M Al Malouf C. Sarcopenia and cardiovascular diseases. Circulation. (2023) 147:1534–53. doi: 10.1161/CIRCULATIONAHA.123.064071, PMID: 37186680 PMC10180053

[2] Sayer AA Cruz-Jentoft A. Sarcopenia definition, diagnosis and treatment: consensus is growing. Age Ageing. (2022) 51:220. doi: 10.1093/ageing/afac220, PMID: 36273495 PMC9588427

[3] Cho M-R Lee S Song S-K. A review of sarcopenia pathophysiology, diagnosis, treatment and future direction. J Korean Med Sci. (2022) 37:e146. doi: 10.3346/jkms.2022.37.e146, PMID: 35535373 PMC9091430

[4] Petermann-Rocha F Balntzi V Gray SR Lara J Ho FK Pell JP. Global prevalence of sarcopenia and severe sarcopenia: a systematic review and meta-analysis. J Cachexia Sarcopenia Muscle. (2022) 13:86–99. doi: 10.1002/jcsm.12783, PMID: 34816624 PMC8818604

[5] Chianca V Albano D Messina C Gitto S Ruffo G Guarino S. Sarcopenia: imaging assessment and clinical application. Abdom Radiol (NY). (2022) 47:3205–16. doi: 10.1007/s00261-021-03294-3, PMID: 34687326 PMC8536908

[6] Sanz-Cánovas J López-Sampalo A Cobos-Palacios L Ricci M Hernández-Negrín H Mancebo-Sevilla JJ. Management of Type 2 diabetes mellitus in elderly patients with frailty and/or sarcopenia. Int J Environ Res Public Health. (2022) 19:8677. doi: 10.3390/ijerph19148677, PMID: 35886528 PMC9318510

[7] Sasaki KI Fukumoto Y. Sarcopenia as a comorbidity of cardiovascular disease. J Cardiol. (2022) 79:596–604. doi: 10.1016/j.jjcc.2021.10.013 34906433

[8] Smith C Woessner MN Sim M Levinger I. Sarcopenia definition: does it really matter? Implications for resistance training. Ageing Res Rev. (2022) 78:101617. doi: 10.1016/j.arr.2022.101617, PMID: 35378297

[9] Chen LK Arai H Assantachai P Akishita M Chew STH Dumlao LC. Roles of nutrition in muscle health of community-dwelling older adults: evidence-based expert consensus from Asian working Group for Sarcopenia. J Cachexia Sarcopenia Muscle. (2022) 13:1653–72. doi: 10.1002/jcsm.12981, PMID: 35307982 PMC9178363

[10] Lyu YH Xie L Chen W Wang J Wei XT Wei YP. Application of metabonomics in study of traditional Chinese medicine syndrome: a review. Zhongguo Zhong Yao Za Zhi. (2022) 47:367–75. doi: 10.19540/j.cnki.cjcmm.20210817.602 35178978

[11] Chan HHL Ng T. Traditional Chinese medicine (TCM) and allergic diseases. Curr Allergy Asthma Rep. (2020) 20:67. doi: 10.1007/s11882-020-00959-9 32875353

[12] Fang Y Luo L Li R. Application of traditional Chinese medicine syndrome differentiation in identification of body constitution of hypertensive and diabetic patients. Am J Transl Res. (2021) 13:12034–42. PMID: 34786139 PMC8581866

[13] Li Z Feiyue Z Gaofeng L. Traditional Chinese medicine and lung cancer--from theory to practice. Biomed Pharmacother. (2021) 137:111381. doi: 10.1016/j.biopha.2021.111381 33601147

[14] Li M Lv X Liu Y Wang L Song J. TCM constitution analysis method based on parallel FP-growth algorithm in Hadoop framework. J Healthc Eng. (2022) 2022:9006096–14. doi: 10.1155/2022/9006096, PMID: 36081755 PMC9448629

[15] Shen T Wang S Wang Z Jia H Wei Y Li Y. Association between the traditional Chinese medicine constitution and metabolic dysfunction-associated fatty liver disease in older people: a cross-sectional study. Heliyon. (2024) 10:e24905. doi: 10.1016/j.heliyon.2024.e24905 38317874 PMC10839603

[16] Mao B Zhao Z Wei M Liu X Zhao R Zhang W. Study on the related factors of TCM constitution and hemodynamics in patients with coronary heart disease. Front Cardiovasc Med. (2024) 11:1383082. doi: 10.3389/fcvm.2024.1383082, PMID: 38529331 PMC10961412

[17] Liang X Wang Q Jiang Z Li Z Zhang M Yang P. Clinical research linking traditional Chinese medicine constitution types with diseases: a literature review of 1639 observational studies. J Tradit Chin Med. (2020) 40:690–702. doi: 10.19852/j.cnki.jtcm.2020.04.019, PMID: 32744037

[18] Shen C Pang SM Kwong EW Cheng Z. The effect of Chinese food therapy on community dwelling Chinese hypertensive patients with Yin-deficiency. J Clin Nurs. (2010) 19:1008–20. doi: 10.1111/j.1365-2702.2009.02937.x, PMID: 20492045

[19] Wang Q Ren XJ Yao SL Wu HD. Clinical observation on the endocrinal and immune functions in subjects with yin-deficiency constitution. Chin J Integr Med. (2010) 16:28–32. doi: 10.1007/s11655-010-0028-9, PMID: 20131033

[20] Yu R Liang J Liu Q Niu XZ Lopez DH Hou S. The relationship of CCL4, BCL2A1, and NFKBIA genes with premature aging in women of Yin deficiency constitution. Exp Gerontol. (2021) 149:111316. doi: 10.1016/j.exger.2021.111316, PMID: 33766622

[21] Chen PH Fang SC Lee SY Lin WL Tsai SF Huang SM. The effect of physical activity on body constitution and psychological health in older adults: evidence from an analysis of a biobank research database. J Aging Phys Act. (2022) 31:465–73. doi: 10.1123/japa.2022-0195, PMID: 36410341

[22] Tandon P Montano-Loza AJ Lai JC Dasarathy S Merli M. Sarcopenia and frailty in decompensated cirrhosis. J Hepatol. (2021) 75:S147–62. doi: 10.1016/j.jhep.2021.01.025, PMID: 34039486 PMC9125684

[23] Jung HN Jung CH Hwang YC. Sarcopenia in youth. Metabolism. (2023) 144:155557. doi: 10.1016/j.metabol.2023.155557 37080353

[24] Jimenez-Gutierrez GE Martínez-Gómez LE Martínez-Armenta C Pineda C Martínez-Nava GA Lopez-Reyes A. Molecular mechanisms of inflammation in sarcopenia: diagnosis and therapeutic update. Cells. (2022) 11:2359. doi: 10.3390/cells11152359, PMID: 35954203 PMC9367570

[25] Williams GR Dunne RF Giri S Shachar SS Caan BJ. Sarcopenia in the older adult with Cancer. J Clin Oncol. (2021) 39:2068–78. doi: 10.1200/JCO.21.00102, PMID: 34043430 PMC8260902

[26] Wen T Liu X Pang T Li M Jiao G Fan X. The efficacy of Chaihu-Guizhi-Ganjiang decoction on chronic non-atrophic gastritis with gallbladder heat and spleen cold syndrome and its metabolomic analysis: an observational controlled before-after clinical trial. Drug Des Devel Ther. (2024) 18:881–97. doi: 10.2147/DDDT.S446336, PMID: 38529263 PMC10962469

[27] Dou Z Xia Y Zhang J Li Y Zhang Y Zhao L. Syndrome differentiation and treatment regularity in traditional Chinese medicine for type 2 diabetes: a text mining analysis. Front Endocrinol. (2021) 12:728032. doi: 10.3389/fendo.2021.728032, PMID: 35002950 PMC8733618

[28] Lv R Zhao Y Wang Z Liu X Wang Z Li S. Obstructive sleep apnea hypopnea syndrome in ancient traditional Chinese medicine. Sleep Breath. (2023) 27:1597–610. doi: 10.1007/s11325-022-02708-w, PMID: 36194363

[29] Papadopoulou SK. Sarcopenia: a contemporary health problem among older adult populations. Nutrients. (2020) 12:1293. doi: 10.3390/nu12051293, PMID: 32370051 PMC7282252

[30] Zhao X Tan X Shi H Xia D. Nutrition and traditional Chinese medicine (TCM): a system's theoretical perspective. Eur J Clin Nutr. (2021) 75:267–73. doi: 10.1038/s41430-020-00737-w, PMID: 32884122

[31] Deng X Teng J Nong X Yu B Tang L Liang J. Characteristics of TCM constitution and related biomarkers for mild cognitive impairment. Neuropsychiatr Dis Treat. (2021) 17:1115–24. doi: 10.2147/NDT.S290692, PMID: 33907404 PMC8068505

[32] Wang J Wong Y-K Liao F. What has traditional Chinese medicine delivered for modern medicine? Expert Rev Mol Med. (2018) 20:e4. doi: 10.1017/erm.2018.3 29747718

[33] Zhao H Ren Q Wang H-Y Zong Y Zhao W Wang Y. Alterations in gut microbiota and urine metabolomics in infants with yin-deficiency constitution aged 0-2 years. Heliyon. (2023) 9:e14684. doi: 10.1016/j.heliyon.2023.e14684, PMID: 37064462 PMC10102239

[34] Chung H-W Tai C-J Chang P Su W-L Chien L-Y. The effectiveness of a traditional Chinese medicine-based Mobile health app for individuals with prediabetes: randomized controlled trial. JMIR Mhealth Uhealth. (2023) 11:e41099. doi: 10.2196/41099, PMID: 37338977 PMC10337399

[35] Zhang Z Chuang Y Ke X Wang J Xu Y Zhao Y. The influence of TCM constitutions and neurocognitive function in elderly Macau individuals. Chin Med. (2021) 16:32. doi: 10.1186/s13020-021-00441-2, PMID: 33849623 PMC8045257

[36] Eisenhardt S Fleckenstein J. Traditional Chinese medicine valuably augments therapeutic options in the treatment of climacteric syndrome. Arch Gynecol Obstet. (2016) 294:193–200. doi: 10.1007/s00404-016-4078-x, PMID: 27040419

[37] Jiang M Lu C Zhang C Yang J Tan Y Lu A. Syndrome differentiation in modern research of traditional Chinese medicine. J Ethnopharmacol. (2012) 140:634–42. doi: 10.1016/j.jep.2012.01.033 22322251

[38] Chia-Yu H Peter Karl M Mei-Yao W Dung-Huan L Pei-Ching W Hung-Rong YJ. The effect of Tai chi in elderly individuals with sarcopenia and frailty: a systematic review and meta-analysis of randomized controlled trials. Ageing Res Rev. (2022) 82:101747. doi: 10.1016/j.arr.2022.101747, PMID: 36223875

[39] Wang J Ma Q Li Y Li P Wang M Wang T. Research progress on traditional Chinese medicine syndromes of diabetes mellitus. Biomed Pharmacother. (2020) 121:109565. doi: 10.1016/j.biopha.2019.109565, PMID: 31704615

