# Supplementary material for: Traditional Chinese medicine constitution and sarcopenia: a cross-sectional study
Source: Front Public Health. 2024 Jul 24;12:1368933. doi: 10.3389/fpubh.2024.1368933 (PMC11304350; doi:10.3389/fpubh.2024.1368933)
Supplement: Supplementary file 1 [file Data_Sheet_1.doc]

**Constitution in Chinese Medicine Questionnaire**

In mainland China, the Constitution in Chinese Medicine Questionnaire (CCMQ) was established by Wang et al. Sixty items were applied to measure the nine constitutions (Table.1-9): Neutral (a normal constitution), Qi-deficiency constitution, Yang-deficiency constitution, Yin-deficiency constitution, Phlegm-dampness constitution, Damp-heat constitution, Blood-stagnation constitution, Qi-stagnation constitution, and Special diathesis constitution.

**Assessing Methods**

Firstly, answer the 60 questions. Secondly, every question is attached to 5 scores and then calculated the total scores and do some adjustments. Finally, estimate which constitution is involved according to the above scores.

Total scores (TS) are scores of every item added up.

**Tables**

**Table1. Yang-deficiency Constitution**

| According to the nearly a year of experience and feeling, please answer the following questions | NO  (not at all) | Scarcely  (little) | Sometimes  (some) | Often  ([comparatively](../../../../D:/Youdao/Dict/6.3.69.8341/resultui/frame/javascript:void(0)%3B)) | Always  (very) |
| --- | --- | --- | --- | --- | --- |
| (1) Did your hands or feet feel cold or clammy? | 1 | 2 | 3 | 4 | 5 |
| (2) Did you feel cold easily in your abdomen, back, lower back or knees? | 1 | 2 | 3 | 4 | 5 |
| (3) Were you sensitive to cold and tend to wear more clothes than others? | 1 | 2 | 3 | 4 | 5 |
| (4) Did you feel more vulnerable to the cold than others(winter coldness, air conditioners, fans, etc.)? | 1 | 2 | 3 | 4 | 5 |
| (5) Did you catch colds more easily than others? | 1 | 2 | 3 | 4 | 5 |
| (6) Did you feel uncomfortable when you drank or ate something cold or do you avoid to drinking or eating something cold? | 1 | 2 | 3 | 4 | 5 |
| (7) Did you easily contract diarrhea when you were exposed to cold or eat(or drink)something cold? | 1 | 2 | 3 | 4 | 5 |
| Results：□Yes   □ Prone to “Yes”   □NO | | | | | |

**Table2. Yin-deficiency Constitution**

| According to the nearly a year of experience and feeling, please answer the following questions | NO  (not at all) | Scarcely  (little) | Sometimes  (some) | Often  ([comparatively](../../../../D:/Youdao/Dict/6.3.69.8341/resultui/frame/javascript:void(0)%3B)) | Always  (very) |
| --- | --- | --- | --- | --- | --- |
| (1)Do the palms of your hands or soles ofyour feet feel hot? | 1 | 2 | 3 | 4 | 5 |
| (2)Did your body and face feel hot? | 1 | 2 | 3 | 4 | 5 |
| (3) Did your skin or lips feel dry? | 1 | 2 | 3 | 4 | 5 |
| (4)Were your lips redder than others? | 1 | 2 | 3 | 4 | 5 |
| (5) Did you get constipated easily or have dry stools? | 1 | 2 | 3 | 4 | 5 |
| (6) Did you get hot flashes? | 1 | 2 | 3 | 4 | 5 |
| (7) Did your eyes feel dry and use eye drops? | 1 | 2 | 3 | 4 | 5 |
| (8)Did you sweat easily when you had a slightly increased physical activity? | 1 | 2 | 3 | 4 | 5 |
| Results：□Yes   □ Prone to “Yes”   □NO | | | | | |

**Table3. Qi-deficiency Constitution**

| According to the nearly a year of experience and feeling, please answer the following questions | NO  (not at all) | Scarcely  (little) | Sometimes  (some) | Often  ([comparatively](../../../../D:/Youdao/Dict/6.3.69.8341/resultui/frame/javascript:void(0)%3B)) | Always  (very) |
| --- | --- | --- | --- | --- | --- |
| (1) Did you get tired easily? | 1 | 2 | 3 | 4 | 5 |
| (2)Did you suffer from shortness of breath? | 1 | 2 | 3 | 4 | 5 |
| (3) Did you get palpitations? | 1 | 2 | 3 | 4 | 5 |
| (4) Did you get dizziness easily or become giddy when standing up? | 1 | 2 | 3 | 4 | 5 |
| (5) Did you catch colds more easily than others? | 1 | 2 | 3 | 4 | 5 |
| (6) Didyou prefer quietness and do not like to talk? | 1 | 2 | 3 | 4 | 5 |
| (7) Do you feel weak when talking? | 1 | 2 | 3 | 4 | 5 |
| (8) Did you sweat easily when you had a slightly increased physical activity? |  |  |  |  |  |
| Results：□Yes   □ Prone to “Yes”   □NO | | | | | |

**Table4. Phlegm-damp Constitution**

| According to the nearly a year of experience and feeling, please answer the following questions | NO  (not at all) | Scarcely  (little) | Sometimes  (some) | Often  ([comparatively](../../../../D:/Youdao/Dict/6.3.69.8341/resultui/frame/javascript:void(0)%3B)) | Always  (very) |
| --- | --- | --- | --- | --- | --- |
| (1) Did you feel chest or stomach stuffiness? | 1 | 2 | 3 | 4 | 5 |
| (2) Did your body feel heavy or lethargic? | 1 | 2 | 3 | 4 | 5 |
| (3) Was your stomach/belly flabby? | 1 | 2 | 3 | 4 | 5 |
| (4) Did you have an excessively oily forehead and/or T—zone? | 1 | 2 | 3 | 4 | 5 |
| (5) Did you have upper eyelid swelling? | 1 | 2 | 3 | 4 | 5 |
| (6) Did your mouth feel sticky? | 1 | 2 | 3 | 4 | 5 |
| (7) Did you have lots of phlegm，especially in your throat? | 1 | 2 | 3 | 4 | 5 |
| (8) Did your tongue have a thick coating? | 1 | 2 | 3 | 4 | 5 |
| Results：□Yes   □ Prone to “Yes”   □NO | | | | | |

**Table5. Damp-heat Constitution**

| According to the nearly a year of experience and feeling, please answer the following questions | NO  (not at all) | Scarcely  (little) | Sometimes  (some) | Often  ([comparatively](../../../../D:/Youdao/Dict/6.3.69.8341/resultui/frame/javascript:void(0)%3B)) | Always  (very) |
| --- | --- | --- | --- | --- | --- |
| (1) Did your nose or your face feel greasy, oily, or shiny? | 1 | 2 | 3 | 4 | 5 |
| (2) Did you get acne or sores easily? | 1 | 2 | 3 | 4 | 5 |
| (3) Did you have bitterness or a strange taste in your mouth? | 1 | 2 | 3 | 4 | 5 |
| (4) Did you pass sticky stools and /or feelthatyour bowel movement is incomplete? | 1 | 2 | 3 | 4 | 5 |
| (5) Did your urethral canal feel hot when you urinated, or did your urine have a dark color? | 1 | 2 | 3 | 4 | 5 |
| (6) Was your vaginal discharge yellowish ly for female interviewees)? | 1 | 2 | 3 | 4 | 5 |
| (7) Was your scrotum always wet(only for male interviewees)? | 1 | 2 | 3 | 4 | 5 |
| Results：□Yes   □ Prone to “Yes”   □NO | | | | | |

**Table6. Blood-stagnation Constitution**

| According to the nearly a year of experience and feeling, please answer the following questions | NO  (not at all) | Scarcely  (little) | Sometimes  (some) | Often  ([comparatively](../../../../D:/Youdao/Dict/6.3.69.8341/resultui/frame/javascript:void(0)%3B)) | Always  (very) |
| --- | --- | --- | --- | --- | --- |
| (1) Did black or purple bruises appear on your skin for no reason? | 1 | 2 | 3 | 4 | 5 |
| (2) Did you have visible capillary/threadveinson yourcheeks? | 1 | 2 | 3 | 4 | 5 |
| (3) Did you feel pain somewhere in your body? | 1 | 2 | 3 | 4 | 5 |
| (4) Did you have a dark face or get brown spots easily? | 1 | 2 | 3 | 4 | 5 |
| (5) Did you get dark circles under the eyes easily? | 1 | 2 | 3 | 4 | 5 |
| (6) Did you forget things easily? | 1 | 2 | 3 | 4 | 5 |
| (7) Did your lips darker, more blue or purpie than usual? | 1 | 2 | 3 | 4 | 5 |
| Results：□Yes   □ Prone to “Yes”   □NO | | | | | |

**Table7. Special diathesisConstitution**

| According to the nearly a year of experience and feeling, please answer the following questions | NO  (not at all) | Scarcely  (little) | Sometimes  (some) | Often  ([comparatively](../../../../D:/Youdao/Dict/6.3.69.8341/resultui/frame/javascript:void(0)%3B)) | Always  (very) |
| --- | --- | --- | --- | --- | --- |
| (1) Did you sneeze even when you did not have a cold? | 1 | 2 | 3 | 4 | 5 |
| (2) Did you have runny or stuffy nose even when you did not have a cold? | 1 | 2 | 3 | 4 | 5 |
| (3) Did you cough due to seasonal change, temperature change, or unpleasant odor? | 1 | 2 | 3 | 4 | 5 |
| 1. Did you have allergies?(E.g. Medicine, food, odors, pollen, pet dander, orduring seasonal or weather change etc.)? | 1 | 2 | 3 | 4 | 5 |
| (5) Did your skin get hives/urticaria easily? | 1 | 2 | 3 | 4 | 5 |
| (6) Did your skin have purpura(purple spots, ecchymosis)due to allergies? | 1 | 2 | 3 | 4 | 5 |
| (7) Did you skin turn red and show traces when you scratched it? | 1 | 2 | 3 | 4 | 5 |
| Results：□Yes   □ Prone to “Yes”   □NO | | | | | |

**Table8. Qi-stagnation Constitution**

| According to the nearly a year of experience and feeling, please answer the following questions | NO  (not at all) | Scarcely  (little) | Sometimes  (some) | Often  ([comparatively](../../../../D:/Youdao/Dict/6.3.69.8341/resultui/frame/javascript:void(0)%3B)) | Always  (very) |
| --- | --- | --- | --- | --- | --- |
| (1) Did you feel gloomy and depressed? | 1 | 2 | 3 | 4 | 5 |
| (2) Do you get anxious and worried easily? | 1 | 2 | 3 | 4 | 5 |
| (3) Did you feel sensitive，vulnerable or emotionally upset? | 1 | 2 | 3 | 4 | 5 |
| (4) Were you easily scared or frightened? | 1 | 2 | 3 | 4 | 5 |
| (5) Did you feel chest or stomach stuffiness? | 1 | 2 | 3 | 4 | 5 |
| (6) Did you sigh for no reason? | 1 | 2 | 3 | 4 | 5 |
| (7) Did your throat feel strange(i.e, likesomething was stuck or there was a lump in your throat)? | 1 | 2 | 3 | 4 | 5 |
| Results：□Yes   □ Prone to “Yes”   □NO | | | | | |

**Table9. Neutral (a normal) constitution**

| According to the nearly a year of experience and feeling, please answer the following questions | NO  (not at all) | Scarcely  (little) | Sometimes  (some) | Often  ([comparatively](../../../../D:/Youdao/Dict/6.3.69.8341/resultui/frame/javascript:void(0)%3B)) | Always  (very) |
| --- | --- | --- | --- | --- | --- |
| (1) Were you energetic? | 1 | 2 | 3 | 4 | 5 |
| (2) Did you get tired easily? | 1 | 2 | 3 | 4 | 5 |
| (3) Do you feel weak when talking? | 1 | 2 | 3 | 4 | 5 |
| (4) Did you feel gloomy and depressed? | 1 | 2 | 3 | 4 | 5 |
| (5) Did you feel more vulnerable to the cold than others (winter coldness, air conditioners, fans, etc.)? | 1 | 2 | 3 | 4 | 5 |
| (6) Could you adapt yourself to external natural or social environment change? | 1 | 2 | 3 | 4 | 5 |
| (7) Did you suffer from insomnia? | 1 | 2 | 3 | 4 | 5 |
| (8) Did you forget things easily? |  |  |  |  |  |
| Results：□Yes   □ Prone to “Yes”   □NO | | | | | |

**Acknowledgement**

As translating the Chinese version of CCMQ, we referred to the English version of CCMQ made by *JING Hui-ru et al*[1]*.*

**Reference:**

JING Hui-ru，WANG Ji，WANG Qi. Preliminary Compiling of English Version of Constitution in Chinese Medicine Questionnaire. *J ANHUI UNIV CHINESE ME* 2015: 34(5): 21-25.
